# A proposed severity classification of borderline symptoms using the borderline symptom list (BSL-23)

**DOI:** 10.1186/s40479-020-00126-6

**Published:** 2020-06-01

**Authors:** Nikolaus Kleindienst, Martin Jungkunz, Martin Bohus

**Affiliations:** 1 Institute of Psychiatric and Psychosomatic Psychotherapy, Central Institute of Mental Health Mannheim, J5, D-68159 Mannheim, Germany / Medical Faculty Mannheim, Heidelberg University, J5, 68159 Mannheim, Germany; 2 National Center for Tumor Diseases, Section for Translational Medical Ethics, Heidelberg University Hospital, Heidelberg, Germany; 3 McLean Hospital, Harvard Medical School, Boston, MA USA

**Keywords:** Assessment, Borderline personality disorder, Illness severity

## Abstract

**Background:**

The Borderline Symptom List (BSL-23) is a well-established self-rating instrument to assess the severity of borderline typical psychopathology. However, a classification of severity levels for the BSL-23 is missing.

**Methods:**

Data from 1.090 adults were used to develop a severity classification for the Borderline Symptom List (BSL-23). The severity grading was based on the distribution of the BSL-23 in 241 individuals with a diagnosis of BPD. Data from three independent samples were used to validate the previously defined severity grades.

These validation samples included a group of treatment seeking patients with a diagnosis of BPD (*n* = 317), a sample of individuals with mental illnesses other than BPD (*n* = 176), and a healthy control sample (*n* = 356). The severity grades were validated from comparisons with established assessment instruments such as the International Personality Disorders Examination, the Structured Clinical Interview for DSM-IV, the global severity index of the Symptom Checklist (GSI, SCL-90), the Global Assessment of Functioning (GAF), and the Beck Depression Inventory (BDI-II).

**Results:**

Six grades of symptom severity were defined for the BSL-23 mean score: none or low: 0–0.28; mild: 0.28–1.07; moderate: 1.07–1.87; high: 1.87–2.67; very high: 2.67–3.47; and extremely high: 3.47–4. These grades received consistent empirical support from the independent instruments and samples. For instance, individuals with a severity grade of *none or low* were virtually free from diagnostic BPD-criteria, had a GSI below the normative population, and a high level of global functioning corresponding to few or no symptoms. Severity grades indicating *high* to *extremely high* levels of BPD symptoms were observed at a much higher rate in treatment-seeking patients (70.0%) than in clinical controls (17.6%) and healthy controls (0.0%). The BSL-23 score that best separated treatment-seeking BPD patients and clinical controls was 1.50, whereas the clearest discrimination of BPD patients and healthy controls was found at a score of 0.64.

**Conclusions:**

The grades of BPD-specific symptom severity derived from the distribution of the BSL-23 scores received consistent empirical validation from established assessments for psychopathology. Future studies should expand this validation by including additional instruments e.g., to assess self-esteem, loneliness, connectedness, and quality of life.

## Background

The categorical diagnosis of borderline personality disorder (BPD) according to the DSM-5 has robust internal and external validity [1]. However, as is common in mental disorders, BPD encompasses a broad range of different symptom characteristics and symptom severity, which is challenging for both treatment and research requirements. For instance, it is unclear whether the currently recommended evidence based psychosocial treatments [2] should be applied in all BPD patients, or whether a differential indication based on symptom severity might reveal better treatment results. In particular, the studies by Bateman & Fonagy [3] and by Sahin et al. [4] indicate that the relative efficacy of treatments might be moderated by the severity of the disorder. Accordingly, most clinicians and researchers agree, that in addition to the categorical diagnostic, the severity of the disorder should be defined. However, as reviewed by Zimmerman et al. [5], there currently is a lack of consensus what exactly is meant by severity of BPD, and how to assess severity of BPD. Marsha Linehan, for example, classifies levels of disorders by the occurrence of current behavior patterns such as self-harm and suicidal acts [6], whereas the DSM-5 Alternative Model for Personality Disorders (AMPD) [7] classifies levels of personality functioning based on self functioning (identity and self-direction) and interpersonal functioning (empathy and intimacy) [8]. While several scales assessing levels of personality functioning as defined in the AMPD have been developed and successfully validated [9–11] these scales are designed for assessing severity of general core factors of personality disorders, not for specifically assessing severity of BPD. The 11th revision of the International Statistical Classification of Disease and related Health Problems (ICD-11) offers another definition of personality disorder severity by determining severity according to emotional, cognitive and behavioral manifestations of “personality dysfunction” such as “the ability and willingness to perform expected social and occupational roles” [12]. This lack of consensus on how to assess BPD severity also includes the definition of cut-offs for different levels of severity of BPD. In contrast to guidelines on the treatment of depressive disorders providing empirically informed guidance for differential treatment of mild, medium, and severe symptomatology [13], research on different levels of symptom severity of BPD is still at quite an early stage. Given the lack of established severity ratings, research studies sometimes revert to diagnostic interviews such as the International Personality Disorders Examination (IPDE), or the Structured Clinical Interview for DSM-5 Personality Disorders (SCID-5-PD) for assessing overall symptom severity in BPD – e.g., by counting the number of diagnostic symptoms as defined in the DSM-5 (APA, 2013) [7]. However, it should be kept in mind that these diagnostic instruments have been designed for establishing diagnoses, and not to assess the severity related to specific diagnoses.

Several instruments have been developed to assess the severity of BPD-specific symptoms. These instruments include both interviews and self-ratings. The current best-established interviews are the Borderline Personality Disorder Severity Index (BPDSI [14]) and the Zanarini Rating Scale for Borderline Personality Disorder (ZAN-BPD [15]). The BPDSI is a semi-structured interview assessing the frequency and severity of BPD during the previous 3 months [14]. It is based on the nine DSM-5 criteria, which assess the frequency of BPD-symptom behaviors on Likert-type items ranging from 0 (never) to 10 (every day). These values can be averaged to yield an overall severity score. The ZAN-BPD is also based on the nine DSM-5 criteria. These items reflect a 1-week time frame and each of the nine criteria for BPD is rated on a five-point anchored rating scale of 0 to 4, yielding a total score of 0 to 36. The ZAN-BPD also exists as a self-rating instrument [16], again based on the nine DSM-5 criteria and yielding a score that reflects the overall severity of BPD-symptoms.

The strict focus on the diagnostic criteria, however, might be at the cost of assessing severity of BPD-symptoms in a comprehensive way and at the cost of sensitivity to change. Beyond the diagnostic criteria stipulated in the DSM-5 and in the ICD-11 patients with a diagnosis of BPD often display additional clinically relevant symptoms such as high levels of shame, guilt, self-contempt, disgust, loneliness and rejection sensitivity [1, 17, 18]. A more comprehensive assessment of clinically relevant BPD-symptoms and sensitivity to change has been among the driving forces that has led to the development of self-rating instruments. Questionnaires that have been specifically designed to assess the severity and the change of severity in BPD-symptoms include the Borderline Evaluation of Severity over Time (BEST) [19], as well as the short and long versions of the Borderline Symptom List (BSL-23, and BSL-95 respectively [20, 21]). The BEST yields a total score, which is based on 15 items that reflect thoughts, feelings and behaviors. This instrument was found to have high internal consistency, discriminant validity, and medium test-retest reliability. The BSL-23 assesses 23 feelings and experiences typically reported by BPD patients, refers to the last week and has a range from 0 = ‘not at all’ to 4 = ‘very strong’. The BSL-23 items are based on criteria of the DSM-IV / DSM-5, on the revised version of the Diagnostic Interview for Borderline Personality Disorder [22], and on the experiences of both clinical experts, and BPD patients who had been included in the scale development. On the one hand, the items cover diagnostic criteria – e.g., affective instability (item 14: “My mood rapidly cycled in terms of anxiety, anger, and depression”), recurrent suicidal behavior, gestures, or threats, or self-mutilating behavior (“I didn’t believe in my right to live”, “The idea of death had a certain fascination for me”, “I thought of hurting myself”, “I wanted to punish myself”), and transient dissociative symptoms (“I felt as if I was far away from myself”). On the other hand, items were added, that are based on borderline-typical empirical findings regarding self-criticism, problems with trust, emotional vulnerability, and proneness to shame, self-disgust, loneliness, and helplessness, e.g., “Criticism had a devastating effect on me”, “I didn’t trust other people”, “I felt vulnerable”). A complete list of all items from the BSL-23 has been published previously [20] and is provided online [23]. The items of the BSL-23 were selected from the 95-item version of the BSL (BSL-95) to define a manageable scale and essentially preserve the comprehensive assessment of BPD-symptoms of the BSL-95. The BSL-23 has one highly dominant eigenvalue that reflects its single factor structure and has good to excellent psychometric properties [21]. These properties have been replicated in several studies that validated the translations of the BSL-23 [24–26] into 18 foreign languages [23]. Both the BEST and BSL-23 were not constructed as diagnostic or screenings instruments; accordingly, they provide no cut-off scores. However, the BEST and BSL-23 were optimized to reflect levels and changes in severity of BPD-symptomatology. A drawback of either scale is the lack of a severity classification that is based on empirical data. Anchored severity ratings, established similarly to the Beck-Depression Inventory [27] and the Childhood Trauma Questionnaire [28], would be highly useful in clinical and scientific settings for interpreting the scores and their changes, i.e., in therapy and (therapy) research.

The aim of the present study was to provide a classification of severity for the BSL-23. As there is no gold-standard for establishing categories of BPD-specific severity and as the BSL-23 was found to be highly homogeneous with a single factor structure, the classification of severity is primarily based on the empirical distribution of BSL-23 mean scores in broadly sampled individuals with a diagnosis of BPD. After establishing this classification from a large sample of BPD patients recruited from various sources, three independent samples were used to provide external validation of the newly established classes: i) a sample of individuals who were applying for a psychological therapy to treat an established diagnosis of BPD, ii) a clinical control sample with individuals with an Axis-I disorder but no diagnosis of BPD, iii) healthy individuals with a documented lifetime absence of BPD. The process of external validation was further complemented by characterizing the severity levels by the number of diagnostic BPD-criteria according to the International Personality Disorder Examination (IPDE [29]). This characterization of severity levels also used an established measure of general severity of psychiatric symptoms – the Global Severity Index (GSI) from the revised version of the Symptom-Check-List (SCL-90-R, [30]) and the well-established Global Assessment of Functioning (GAF, [31]). Finally, the ability of the BSL-23 and its newly defined severity classes to discriminate BPD-patients from healthy and clinical controls was calculated using the areas under the curve obtained from Receiver Operator Curves (ROCs). Additionally, sensitivities, specificities, and Youden-indices were calculated at various levels of BSL-23 mean-scores.

## Methods

### Participants

The participants in this study belonged to four mutually exclusive groups: i) A calibration sample of individuals diagnosed with BPD (BPD_CAL), ii) a validation sample of individuals with a diagnosis of BPD (BPD_VAL), iii) a mixed sample of psychiatric patients, and iv) a group of mentally healthy controls.

The calibration sample (BPD_CAL) was recruited by the Clinical Research Unit 256 (KFO256). It consisted of 241 participants (201 females, 40 males) that met the criteria for BPD who were participating in different studies within the KFO256. These participants were recruited between 2012 and 2018 via BPD platforms on the internet, advertising on flyers and on the project’s and related websites. Participants from all over Germany took part in the study which was based in Mannheim and Heidelberg, Germany. The mean age in this group was 29.43 (SD: 8.15; range: 18–50).

The validation sample (BPD_VAL) was recruited between 2002 and 2008 in the Department of Psychiatry and Psychotherapy at the University of Freiburg, Germany and at the Central Institute for Mental Health in Mannheim, Germany. This sample sample comprised 317 treatment seeking women with an established diagnosis of BPD. Mean age was 28.52 (SD: 7.91; range: 18–55).

The sample of clinical controls (CC) was recruited at the Psychiatric Hospital at the University of Freiburg, Germany between 2000 and 2001 [21, 32]. This sample consisted of 176 individuals (107 female, 69 male, mean age: 41.44 ranging from 18 to 77) with different axis I disorders (schizophrenia: *n* = 46, delusional disorder: *n* = 3, major depressive disorder: *n* = 75, other affective disorders: *n* = 6, anxiety disorder: *n* = 17, obsessive compulsive disorder: *n* = 27, eating disorder: *n* = 2).

Participants from the healthy control sample (HC) were recruited within the KFO256 between 2012 and 2018 via flyers, websites inviting healthy individuals to participate in a clinical study, and from the residents’ registration office in Mannheim, Germany. This sample consisted of 356 participants (282 female, 74 male) with no psychiatric disorder (lifetime) and with a mean age of 27.68 (SD: 6.88; range: 18–55).

### Instruments

The diagnosis of BPD or the absence of the diagnostic criteria of BPD was established using the IPDE [29]. Axis-I disorders were diagnosed using the German version of the Structured Clinical Interview for the DSM-IV (SCID [33]). The diagnostic interviews and, in addition, the Global Assessment of Functioning (GAF [31]) in the four samples were conducted by trained clinicians.

In both the calibration sample (BPD_CAL) and in the sample of healthy controls (HC), the 23 items pertaining to the BSL-23 were assessed directly from the BSL-23. In the BPD_VAL and CC samples, the BSL-23 was extracted from the 95 items assessed using the BSL-95. Mean scores of the BSL-23 were calculated for those participants who filled in at least 21 out of the 23 questions on the BSL-23 (respectively, the 23 pertinent items of the BSL-95).

Patients from the BPD_CAL and HC samples also completed the Symptom Checklist 90 (SCL-90) [30], which was used to calculate the Global Severity Index (GSI).

### Procedures

The severity classification of the BSL-23 mean score was based on the distribution in the calibration sample (BPD_CAL) of participants with a BPD diagnosis. The distribution was characterized by its mean, standard deviation, skewness, and kurtosis (i.e., the excess kurtosis as defined by Snedecor and Cochran [34]). Normality of this distribution was evaluated using a formal test (Anderson and Darling [35]) and by visual examination of the Q-Q plots displaying the observed quantiles against the quantiles expected under the assumption of normality. The mean and standard deviation were used to establish 6 distribution-based categories of severity: (i) *Extremely high*: BSL-23 mean scores greater than two standard deviations above the mean; (ii) *very high*: scores between one and two standard deviations above the mean; (iii) *high*: scores between the mean and one standard deviation above the mean; (iv) *moderate*: scores between the mean and one standard deviation below the mean; (v) *mild*: scores between one and two standard deviations below the mean; (vi) *none or low*: BSL-23 mean values from 0 to less than two standard deviations below the mean.

### Statistical analyses

Because the categories of severity are on an ordinal scale, ordinal correlations (according to Spearman) were used. The effect-size interpretation of correlations followed the established standards [36], i.e., correlations ≥0.5 or ≤ − 0.5 were considered large; the respective thresholds for medium and small effect-sizes were set at 0.3 and 0.1, respectively. Differences between dependent correlations with one variable in common were tested using the procedure described by Steiger [37]. Finally, receiver operating curves (ROCs) were used to evaluate the sensitivity and specificity of the BSL-23, and of the BSL-23 severity classes to correctly identify the presence or absence of a diagnosis of BPD at different cut-offs. The discriminating capacity of the BSL-23 and of the BSL-23 severity classes at different cut-offs was evaluated using the Youden index, which is defined as sensitivity + specificity - 1. The overall capacity of the BSL-23 and of the BSL-23 severity classes was evaluated by the area under the curve (ROC AUC). Following the recommendations by Streiner and Cairney [38], ROC AUC values between 0.5 and 0.7, between 0.7 and 0.9, and values between 0.9 and 1 indicated low, medium and high discriminating accuracy, respectively. Two-tailed *p*-values ≤0.05 were considered statistically significant. ROC analyses were carried out using SPSS™ (v.20). Tests for comparing correlations were carried out using a program by Lee and Preacher [39]. All other statistical calculations and analyses were carried out using SAS™ v. 9.4.

## Results

**Fig. 1:**
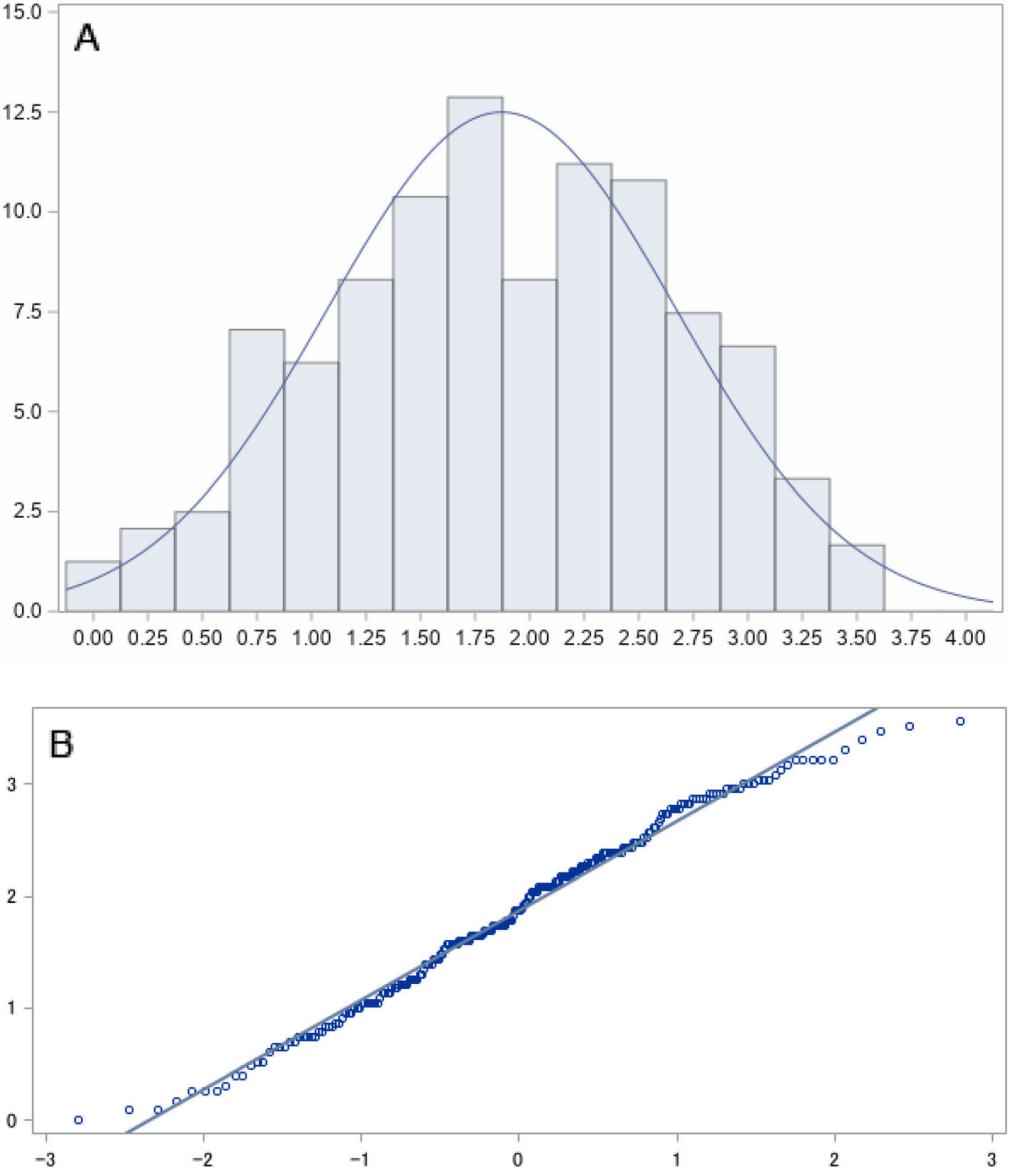
Distribution of the BSL-23 score in *n* = 241 participants with a diagnosis of BPD. **a** Histogram displaying the distribution of BSL-23 mean scores. **b** Q-Q plots displaying the observed quantiles against the quantiles expected under the assumption of normality

The mean score of the BSL-23 in the sample of 241 patients that had a current diagnosis of BPD (BPD_CAL) was 1.87, with a standard deviation (SD) of .8. As illustrated in Fig. 1, the distribution of the BSL-23 scores was approximately symmetrical around the mean (see histogram), and did not significantly deviate from normality (cf. Q-Q plot and histogram). Accordingly, the skewness and the (excess) kurtosis were close to zero (skew = − 0.14, kurt = − 0.67), and despite the relatively large sample size, the Anderson-Darling test did not indicate a significant deviation from normality (A-Sq = 0.75, *p* = 0.05).

Based on the distribution of the BSL-23 mean scores in the calibration sample (BPD_CAL), the dimensional scores were divided into 6 grades of severity: (i) *none or low*: BSL-23 mean scores from 0 (i.e., the theoretical minimum) to less than .28 (i.e., 2 SDs below the mean); (ii) *mild*: scores between .28 and 1.07 (i.e., 1 SD below the mean); (iii) *moderate*: scores between 1.07 and 1.87 (i.e., the mean); (iv) *high*: scores from 1.87 to 2.67 (i.e., 1 SD above the mean); (v) *very high*: scores from 2.67 to 3.47 (i.e., 2 SDs above the mean); (vi) *extremely high*: from 3.47 to 4 (i.e., the theoretical maximum).

Correlations of the BPD severity grades with theoretically convergent measurements mostly conformed to theory. In the combined sample, including all of *n* = 1.090 participants from the four groups (BPD_CAL, BPD_VAL, HC, and CC), the correlation between the BPD severity grades and the original BSL-23 scores was rho = 0.98 (*p* < 0.001). In the combined subsamples of *n* = 558 participants from the two groups with a diagnosis of BPD (BPD_CAL and BPD_VAL), this correlation was still as large as rho = 0.97 (*p* < 0.001). The correlation between the BPD severity grades and the number of BPD-symptoms (as assessed by the IPDE) was available in 908 participants from the BPD_CAL, BPD_VAL, and HC samples. In these 908 samples this correlation was large (rho = 0.80, *p* < 0.001); however, in the subsample that included all BPD patients (BPD_CAL and BPD_VAL, *n* = 558), the correlation was only small to medium (rho = 0.23, *p* < 0.001). The correlation between BPD severity grades and GAF showed a similar pattern. While this correlation was large (rho = -0.80, *p* < 0.001) in the combined sample of BPD and HC participants (BPD_CAL and HC, *n* = 588), the correlation was only small to medium (rho = -0.27, *p* < 0.001) in the BPD patients (BPD_CAL, *n* = 235). Conversely, the correlation between BPD severity grades and the global severity index (GSI) was large in the sample that included all available BPD patients (rho = 0.77, *p* < 0.001). 

In summary, the extremely large positive correlations of the BPD severity grades and the original BSL-23 suggest that the loss of information related to the grouping is minor. The pattern of correlations between the BPD severity grades and theoretically convergent measures also suggests that that BSL-23 severity grades assess psychopathology that is not included when using a very narrow assessment of BPD-symptoms, such as the number of BPD-symptoms (defined in the DSM-5), or when using a rather unspecific measure such as the GAF. The pattern of correlations further supports the convergent validity of the BSL-23 severity grades. Validity of the BPD severity grades is also supported by the cross-tabulation of the 6 classes established in the calibration sample (BPD_CAL) and the means ± standard deviations of the above mentioned external measures (i.e., the number of BPD-symptoms, the GSI, and the GAF) in the combined sample of all BPD and HC participants (BPD_CAL, BPD_VAL, and HC, *n* = 914). As presented in Table 1, the mean values of these external measures suggest a rather functional level in individuals from the two lowest BSL-23 classes (*none or low*, and *mild*). Individuals from the class labelled *none or low* are virtually free from BPD-symptoms as defined in the DSM-5, and were typically found to have a global severity index (GSI) of 0.10, which is below the mean of 0.31 in the mixed adult norm sample described by Franke et al. [40]. These individuals also presented a GAF of 89.62, which (by definition) corresponds to a high level of global functioning and few or no symptoms [31]. According to the variables used for validating the BSL-23 grades, individuals with a severity classification of BPD-symptoms labelled *mild* show some symptoms. The mean number of 3.93 ± 3.10 BPD-symptoms indicate that the majority of participants do not meet the diagnostic criteria for BPD. However, a minority of these individuals do reach the threshold for 5 diagnostic criteria, which qualify these individuals for a diagnosis of BPD. Similarly, a mean of 0.58 in the GSI is just below the value of 0.62, which indicates the threshold for caseness according to Franke [40] and the mean of 67.79 in the GAF falls into the category indicating “no more than slight impairment”. As further shown in Table 1, individuals from the BSL-23 classes *moderate, high, very high*, and *extremely high* all have mean number of 6 or more diagnostic criteria for BPD and mean GSI-scores clearly indicating a need for psychiatric treatment and moderate to serious symptoms according to the mean GAF-values. The BSL-23 classes ranging from *moderate* to *extremely high* showed clear differentiation with respect to both the number of BPD-criteria (for details see Table 1) and with respect to GSI-scores. The observed mean of 1.17, with respect to the GSI-score in the class labelled *moderate* corresponds to the mean GSI-scores of 1.19–1.32 observed in mixed samples of adults receiving psychiatric treatment (Franke [40]), while the mean scores of 1.68, 2.01, and 2.91 observed in the BSL-23 classes labelled *high*, *very high*, and *extremely high*, respectively, also correspond to high, very high, and extremely high mean values in the GSI. In contrast, the four highest BSL-23 classes showed little differentiation with respect to the GAF (for details see Table 1).

**Table 1:** Means and SD of the number of BPD-symptoms, the GSI, and the GAF in every category of severity for BPD_CAL, BPD_VAL, and HC

BSL-23 in the BPD calibration sample (BPD_CAL)		Values of external measures (BPD and HC samples) across the BSL-23 classes of severity		
Severity classification (BSL-23)	Range of BSL-23 mean scores	Number of BPD-symptoms (IPDE)	Global Severity Index (GSI, SCL-90-R)	Global Assessment of Functioning (GAF)
None or low	0–.28 rounded: [0, .3)	.17 ± .98	.10 ± .0.10	89.62 ± 8.76
Mild	.28–1.07 rounded: [.3, 1.1)	3.93 ± 3.10	.58 ± .30	71.55 ± 17.65
Moderate	1.07–1.87 rounded: [1.1, 1.9)	6.29 ± 1.24	1.17 ± .35	52.99 ± 8.01
High	1.87–2.67 rounded: [1.9, 2.7)	6.58 ± 1.25	1.68 ± .45	50.82 ± 8.59
Very high	2.67–3.47 rounded: [2.7, 3.5)	6.99 ± 1.38	2.01 ± .48	48.45 ± 7.78
Extremely high	3.47–4 rounded: [3.5, 4]	7.06 ± 1.26	2.91 ± .13	55.00 ± 5.29

In order to further characterize the BPD severity levels, two additional analyses implying the 23 items of the BSL-23 have been carried out. First, to provide a more nuanced differentiation within the lower end of severity levels (i.e. those ranging from severity level 1 (none or low) to severity level 3 (moderate)), the level (range: 1 to 3) was correlated with each of the BSL-23 items. Second, to study symptomatic differentiation within the higher end of severity levels (i.e. those ranging from 4 (high) to 6 (extremely high)) these levels (range: 4 to 6) were again correlated to all of the items from the BSL-23. These post-hoc analyses were applied to the combined sample of all BPD patients (BPD_CAL and BPD_VAL) and carried out using Spearman correlations (rho) which were classified as “large effect sizes” if rho was equal or larger than 0.5 (Cohen, 1988). Within the lower range of severity classes effect-sizes were large for two items: i) for the item “I hated myself”, rho = 0.55, and for the item "My mood rapidly cycled in terms of anxiety, anger, and depression", rho = 0.54). On the higher end, the following items were strongly related (i.e. rho was at least 0.5) with the level of severity (now ranging from 4 (high) to 6 (extremely high)):“I didn’t believe in my right to live” (rho = 0.56), "I hated myself”(rho = 0.55), “The idea of death had a certain fascination for me” (rho = 0.50), and “I wanted to punish myself” (rho = 0.56). When also considering the four items for which the effect-size was approaching a large effect (i.e. between 0.45 and 0.49) (“I thought of hurting myself”, “I felt disgusted by myself”, “I felt worthless", and “everything seemed senseless to me”), a consistent interpretation is getting obvious: A clinically important feature differentiating those BPD patients with a high over very high to extremely high symptomatic level relates to potentially dangerous self-damaging action tendencies that are grounded in self-contempt and self-hatred.

The validity of the BSL-23 classes was further supported by a cross-tabulation of the severity classes against the samples investigated in this study. In the group of healthy controls (HC), 89% showed *none or low* symptoms, while 11% showed *mild* symptoms. In contrast, the majority (70%) of treatment seeking patients with a diagnosis of BPD (BPD_VAL) presented with a *high, very high* or *extremely high* severity of BPD-symptoms (for details see Table 2). In the sample of clinical controls, the mode (44%) was in the category of *mild* symptoms followed by the adjacent categories (i.e., *moderate*: 24% and *none or low*: 15%). A complete list of percentiles of BSL-23 mean scores in the four subsamples investigated in this study is provided in supplementary table 1.

**Table 2:** Means, standard deviations, and percentiles of BSL-23 mean scores

Sample	N	BSL-23 Mean ± SD	Severity classification (BSL-23)					
			*none or low*	*mild*	*moderate*	*high*	*very high*	*extremely high*
BPD_CAL	241	1.87 ± 0.8	2.90%	15.77%	31.95%	30.71%	17.43%	1.24%
BPD_VAL	317	2.34 ± 0.86	0.63%	9.15%	20.19%	31.55%	29.65%	8.83%
CC	176	1.08 ± 0.79	14.77%	43.75%	23.86%	13.07%	4.55%	0.00%
HC	356	0.12 ± 0.17	88.76%	10.96%	0.28%	0.00%	0.00%	0.00%

**Fig. 2:**
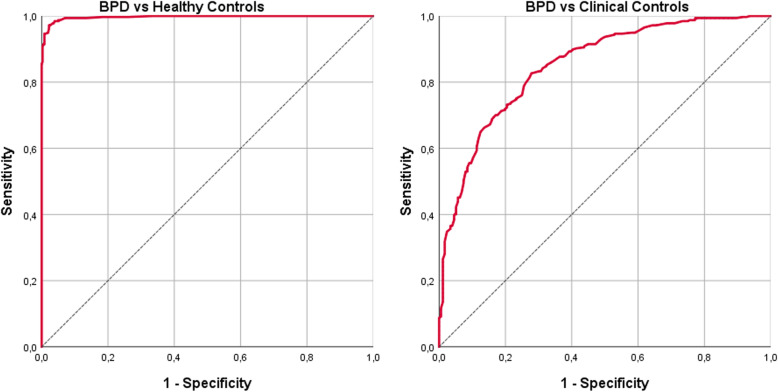
ROC curves displaying the accuracy of the BSL-23 in correctly classifying the diagnostic status of participants (BPD: yes/no) in two samples. Left: In the combined sample of *n* = 317 BPD patients from the BPD_VAL sample and the *n* = 356 healthy controls from the HC sample; Right: in the combined sample of *n* = 317 BPD patients from the BPD_VAL sample and the *n* = 176 clinical controls from the CC sample

The accuracy of the BSL-23 in correctly identifying the diagnostic status (BPD yes vs. no as diagnosed by clinical psychologists using the IPDE) is displayed in Fig. 2. As illustrated in the receiver operator curve (ROC) to the left, the BSL-23 was very accurate in discerning BPD patients from healthy individuals. Accordingly, the area under the curve (ROC AUC) for the BSL-23 mean scores and for the BSL-23 severity classes were as high as 0.997 and 0.991, respectively. The Youden index reached 0.95 with the highest values in the BSL-23 severity class indicating *mild* symptoms (at a BSL-23 mean score of 0.64) where the simultaneous pair of sensitivity and specificity was 0.97 and 0.98, respectively. For a complete list of sensitivities and specificities at all BSL-23 mean scores, see supplementary table 2. As illustrated in the ROC curve to the right of Fig. 2, the BSL-23 also separated BPD patients from individuals with a diagnosis of a mental disorder other than BPD. The area under the curve in this sample was 0.85 for the BSL-23 mean scores and 0.84 for the BSL-23 severity categories, indicating a medium to high level of accuracy in both cases. The Youden index reached 0.55, with the highest values in the BSL-23 severity class indicating *moderate* symptoms (at a BSL-23 mean score of 1.5) where the simultaneous pair of sensitivity and specificity was 0.83 and 0.72, respectively. The complete table of sensitivities and specificities in BPD vs. clinical controls for all values of the BSL-23 is displayed in the supplementary table 3.

## Discussion

The present study aimed to provide an evidence-based severity classification for the 23-item version of the Borderline Symptom List (BSL-23). Based on the distribution of the BSL-23 in 241 individuals with an established diagnosis of BPD, six grades of severity were defined: (i) *None or low*, (ii) *mild*, (iii) *moderate*, (iv) *high*, (v) *very high*, and (vi) *extremely high*. This classification was validated using independent samples (total *n* = 849) and by established instruments that assess the number of BPD-criteria, the global symptom burden and from the Global Assessment of Functioning (GAF) in patients with mental disorders. The cut-offs and labels of the BSL-23 categories *none or low* and *mild* received validation from the distribution in the subsample of healthy controls, with 89 and 11% showing *none or low* and *mild* symptoms, respectively. Participants across all diagnostic groups who were categorized into the *none or low* BPD-symptoms group (according to the BSL-23) were virtually free from diagnostic criteria for BPD, showed a global severity index (GSI) well below the mean of the adult normative population and were found to have a high level of functioning that corresponded with few or no symptoms [31]. These findings provided further evidence for the validity of the first BSL-23 category. According to the interviews and questionnaires used in the validation process, participants categorized as *mild* according to the BSL-23 classification consistently showed some symptoms. However, the mean levels of disorder-specific and general symptom burden did not quite reach the level of caseness, and the GAF indicated “no more than slight impairment”, which further supports the validity of the cut-offs and labelling used in the second BSL-23 category. In addition, individuals from this BSL-23 category were typically found to have significant distress and impairment according to the external validation criteria. The BSL-23 categories four to six, i.e. those ranging from *high* over *very high* to *extremely high* showed a small increase in the mean number of BPD-criteria (from 6.6 to 7.1), and a large increase with respect to the mean GSI (from 1.7 to 2.9). The majority of treatment-seeking patients (70%) fell into one of these three highly symptomatic categories, thus providing further support for the clinical validity of the underlying categorization. Additional exploratory analyses provided preliminary evidence that dangerous self-damaging action tendencies, possibly related to particularly high levels of self-contempt and self-hatred, are characteristic for the highest symptomatic categories. In summary, the six grades of severity as defined by the distribution of the BSL-23 in the calibration sample of BPD-patients, were successfully validated by independent samples and instruments. The validation was supplemented by analyses that were applied to the complete set of severity classes. In a ROC analyses applied to the independent samples, the BSL-23 severity grades clearly differentiated treatment-seeking BPD patients from both healthy controls and from clinical controls with ROC AUCs of 0.99 and 0.84, respectively. These findings are in support of the sensitivity and specificity of the BSL-23 severity grades. Finally, both ROC and correlational analyses revealed that the loss of information was minor when the BSL-23 mean scores are substituted by BSL-23 severity grades. The correlational analyses further indicated that the BSL-23 severity grades assess psychopathology that is not captured when using a narrow assessment of BPD-symptoms, such as the number of BPD-symptoms that are defined in the DSM-5.

While graded assessments of disorder specific severity are available for various psychiatric disorders and syndromes (e.g., the Beck Depression Inventory for assessing depressive symptoms [27] and the Young Mania Rating Scale for assessing manic symptoms [41]), no questionnaire that provides anchored ratings has been available to assess the severity of BPD. Given the consistent and plausible results from the external validation of these BSL-23 severity grades, the findings from the present study clearly extend the clinical utility of the BSL-23, which is a widely used instrument for assessing symptoms typically encountered in BPD-patients.

Our study has both strengths and limitations. A strength of the study is its use of structured clinical interviews administered by specifically trained assessors to determine the diagnostic status. The diagnostic interviews were used to establish both the presence and absence of a BPD diagnosis, thus providing a high level of diagnostic accuracy. Second, the sample size of 1.090 participants is quite large and included 849 participants who contributed to the evaluation of the results from the calibration sample. It should be mentioned, though – and this presents a limitation – that not all of the participants were assessed using the full range of questionnaires and observer-based assessments that were used for the validation process. However, the subgroup of participants who were assessed using the full set of assessment instruments used in the validation process was still quite large (*n* = 585). A second limitation relates to cross-sectional nature of the assessments. Accordingly, it was not possible to assess the sensitivity to change of the BSL-23 severity grades or to empirically evaluate the clinically important question regarding the extent the BSL-23 severity classes are useful in guiding clinical decisions, such as the initiation of treatment or the choice between treatment options. However, the original version of the BSL-23 has shown that it is highly sensitive to change, both in the original validation study [21] and in subsequent treatment studies (e.g., [42]). Because of the very high correlation of the BSL-23 severity classification and the original version of the BSL-23 (rho = 0.97 to 0.98 in the samples investigated in our study), it seems likely that the BSL-23 severity classes will show sensitivity to change in treatment studies. Third, we would like to emphasize that the phenomenology of BPD is too complex to be summarized in six severity classes. Accordingly, a more comprehensive assessment battery is needed to provide a more complete assessment of the patients’ problems related to BPD. In particular, assessments reflecting the subjective experiences of the patients, including quality of life and aspects of well-being such as self-esteem, a positive evaluation of the own body [43], and connectedness [44, 45] would add value and clinical meaning to the classes of symptom severity. Fourth, it has to be acknowledged, that the definition of cut-offs in an approximately normally distributed dimensional assessment remains somewhat arbitrary. Given the lack of an established method, alternative methods for setting the cut-offs and for labelling the severity grades might be equally or more advantageous. Fifth, the potentially important finding that potentially dangerous self-damaging action tendencies are characteristic for the highest symptomatic categories resulted from post-hoc analyses and thus requires replication from an independent study. Finally, the reader should keep in mind that the accuracy in discriminating individuals between different diagnostic groups depends on the specific diagnoses, on the setting, and on additional characteristics of the individuals from these diagnostic groups.

## Conclusion

We propose an empirically based severity-classification for one of the most widely used assessments of BPD-specific symptoms. External validation from three independent samples and from established assessments of psychopathology provided consistent support for a graduated classification of severity ranging from *none or low* to *extremely high*. In addition, this study confirms that BSL-23 is able to discriminate clinical and healthy controls in cohorts of patients with a diagnosis of BPD. A *moderate* symptom level (BSL-23 mean scores of 1.50) was found to be best suited for discriminating BPD patients from a group of mixed clinical controls, whereas a *mild* symptom level (BSL-23 mean scores of 0.64) yielded the best separation of individuals with a diagnosis of BPD from healthy controls. Future research should expand the validation of borderline symptom severity grades by including additional scales to assess important aspects such as self-esteem, connectedness, rejection-sensitivity, impulsivity, and loneliness. Inclusion of further BPD-specific features will provide a more comprehensive characterization of BPD-severity grades.

## Supplementary information

**Additional file 1: Table S1.** Complete list of percentiles of BSL-23 mean scores.

**Additional file 2: Table S2.** Youden’s Index and Coordinates of the ROC curve for BPD_VAL vs HC.

**Additional file 3: Table S3.** Youden’s Index and Coordinates of the ROC curve for BPD_VAL vs CC.

## Data Availability

The data analyzed for the present study are available from the corresponding author on reasonable request.

## Abbreviations

AUC: Area under the curve
BDI: Beck depression inventory
BEST: Borderline evaluation of severity over time
BPD: Borderline personality disorder
BPD_CAL: Calibration sample including patients with a diagnosis of borderline personality disorder
BPD_VAL: Validation sample including patients with a diagnosis of borderline personality disorder
BPDSI: Borderline personality disorder severity index
BSL: Borderline symptom list
BSL-23: Borderline symptom list (short version)
CC: Clinical control sample
CTQ: Childhood trauma questionnaire
DSM-5: Diagnostic and statistical manual, 5th edition
DSM-IV: Diagnostic and statistical manual, 4th edition
GAF: Global assessment of functioning
GSI: Global severity index
HC: Healthy controls
ICD-11: International classification of diseases, 11th revision
IPDE: International personality disorder examination
KFO256: Clinical research unit 256
Kurt: Kurtosis
Q-Q plot: Quantil-Quantil plot
ROC: Receiver operator curve
SCID-I: Structured clinical interview for DSM-IV
SD: Standard deviation
Skew: Skewness
ZAN-BPD: Zanarini rating scale for borderline personality disorder
