# Correction to: A proposed severity classification of borderline symptoms using the borderline symptom list (BSL-23)

**DOI:** 10.1186/s40479-021-00174-6

**Published:** 2022-01-21

**Authors:** Nikolaus Kleindienst, Martin Jungkunz, Martin Bohus

**Affiliations:** 1 Institute of Psychiatric and Psychosomatic Psychotherapy, Central Institute of Mental Health Mannheim, J5, D-68159 Mannheim, Germany; 2 Medical Faculty Mannheim, Heidelberg University, J5, 68159 Mannheim, Germany; 3 National Center for Tumor Diseases, Section for Translational Medical Ethics, Heidelberg University Hospital, Heidelberg, Germany; 4 McLean Hospital, Harvard Medical School, Boston, MA USA

**Correction to: bord personal disord emot dysregul 7, 11 (2020)**

**https://doi.org/10.1186/s40479-020-00126-6**

Following publication of this article [1], it is reported that this article contained some errors.

In Table 1, there are two errors:

1. ‘rounded: [1.1–1.7)’ should be corrected to ‘rounded: [1.1–1.9)’
2. ‘rounded: [1.7–2.7)’ should be corrected to ‘rounded: [1.9–2.7)”

The correct Table 1 is presented in this Correction.

**Table 1:** Means and SD of GAF and GSI in every category of severity for BPD_CAL and HC

BSL-23 in the BPD calibration sample (BPD_CAL)		Values of external measures (BPD and HC samples) across the BSL-23 classes of severity		
Severity classification (BSL-23)	Range of BSL-23 mean scores	Number of BPD-symptoms (IPDE)	Global Severity Index (GSI, SCL-90-R)	Global Assessment of Functioning (GAF)
None or low	0–.28 rounded: [0, .3)	.19 ± .95	.11 ± .0.11	88.35 ± 10.5
Mild	.28–1.07 rounded: [.3, 1.1)	2.88 ± 2.76	.62 ± .3	67.79 ± 15.57
Moderate	1.07–1.87 rounded: [1.1, 1.9)	5.22 ± 2.04	1.17 ± .37	53.69 ± 9.55
High	1.87–2.67 rounded: [1.9, 2.7)	5.90 ± 1.75	1.62 ± .46	50.51 ± 9.61
Very high	2.67–3.47 rounded: [2.7, 3.5)	6.5 ± 1.73	1.99 ± .44	47.49 ± 8.5
Extremely high	3.47–4 rounded: [3.5, 4]	7.2 ± 1.23	2.76 ± 0.39	49.8 ± 10.71

In Abstract section, the sentence below needs correction:

‘none or low: 0–0.3; mild: 0.3–0.7; moderate: 0.7–1.7; high: 1.7–2.7; very high: 2.7–3.5; and extremely high: 3.5–4.’ should be corrected to ‘none or low: [0–0.28); mild: [0.28–1.07); moderate: [1.07–1.87); high: [1.87–2.67); very high: [2.67–3.47); and extremely high: [3.47–4].’

The original article has been updated.
