# Supplementary material for: A proposed severity classification of borderline symptoms using the borderline symptom list (BSL-23)
Source: Borderline Personal Disord Emot Dysregul. 2020 Jun 1;7:11. doi: 10.1186/s40479-020-00126-6 (PMC7262769; doi:10.1186/s40479-020-00126-6)
Supplement: Supplementary file 1 — Additional file 1: Table S1. Complete list of percentiles of BSL-23 mean scores. [file 40479_2020_126_MOESM1_ESM.docx]

Supplementary table 1: Complete list of percentiles of BSL-23 mean scores.

| **Percentiles** | **BPD_CAL** | **BPD_VAL** | **CC** | **HC** |
| --- | --- | --- | --- | --- |
| 1**%** | .087 | .391 | .000 | .000 |
| 2**%** | .261 | .522 | .043 | .000 |
| 3**%** | .304 | .652 | .080 | .000 |
| 4**%** | .391 | .696 | .087 | .000 |
| 5**%** | .522 | .696 | .087 | .000 |
| 6**%** | .652 | .826 | .087 | .000 |
| 7**%** | .652 | .913 | .130 | .000 |
| 8**%** | .739 | .913 | .174 | .000 |
| 9**%** | .739 | 1.000 | .217 | .000 |
| 10**%** | .783 | 1.087 | .217 | .000 |
| 11**%** | .826 | 1.174 | .261 | .000 |
| 12**%** | .826 | 1.217 | .261 | .000 |
| 13**%** | .913 | 1.261 | .261 | .000 |
| 14**%** | .957 | 1.304 | .261 | .000 |
| 15**%** | 1.000 | 1.366 | .304 | .000 |
| 16**%** | 1.043 | 1.435 | .304 | .000 |
| 17**%** | 1.043 | 1.478 | .304 | .000 |
| 18**%** | 1.043 | 1.522 | .304 | .000 |
| 19**%** | 1.087 | 1.565 | .304 | .000 |
| 20**%** | 1.130 | 1.565 | .320 | .000 |
| 21**%** | 1.174 | 1.609 | .348 | .000 |
| 22**%** | 1.217 | 1.652 | .348 | .000 |
| 23**%** | 1.217 | 1.652 | .435 | .000 |
| 24**%** | 1.217 | 1.696 | .435 | .000 |
| 25**%** | 1.261 | 1.701 | .476 | .000 |
| 26**%** | 1.261 | 1.739 | .478 | .000 |
| 27**%** | 1.348 | 1.783 | .522 | .000 |
| 28**%** | 1.391 | 1.798 | .522 | .000 |
| 29**%** | 1.391 | 1.870 | .565 | .000 |
| 30**%** | 1.435 | 1.913 | .565 | .000 |
| 31**%** | 1.478 | 1.913 | .589 | .000 |
| 32**%** | 1.522 | 1.957 | .609 | .000 |
| 33**%** | 1.565 | 2.000 | .609 | .000 |
| 34**%** | 1.565 | 2.043 | .609 | .043 |
| 35**%** | 1.609 | 2.043 | .624 | .043 |
| 36**%** | 1.609 | 2.087 | .652 | .043 |
| 37**%** | 1.609 | 2.130 | .652 | .043 |
| 38**%** | 1.652 | 2.130 | .652 | .043 |
| 39**%** | 1.652 | 2.130 | .696 | .043 |
| 40**%** | 1.652 | 2.174 | .696 | .043 |
| 41**%** | 1.670 | 2.217 | .697 | .043 |
| 42**%** | 1.696 | 2.217 | .711 | .043 |
| 43**%** | 1.696 | 2.261 | .739 | .043 |
| 44**%** | 1.739 | 2.261 | .739 | .043 |
| 45**%** | 1.739 | 2.304 | .754 | .043 |
| 46**%** | 1.739 | 2.348 | .783 | .043 |
| 47**%** | 1.783 | 2.348 | .783 | .043 |
| 48**%** | 1.783 | 2.391 | .826 | .043 |
| 49**%** | 1.870 | 2.391 | .826 | .043 |
| 50**%** | 1.870 | 2.407 | .848 | .043 |
| 51**%** | 1.885 | 2.435 | .870 | .043 |
| 52**%** | 1.957 | 2.435 | .913 | .043 |
| 53**%** | 2.000 | 2.435 | .920 | .043 |
| 54**%** | 2.043 | 2.478 | .928 | .087 |
| 55**%** | 2.087 | 2.522 | .957 | .087 |
| 56**%** | 2.087 | 2.522 | 1.000 | .087 |
| 57**%** | 2.087 | 2.565 | 1.043 | .087 |
| 58**%** | 2.087 | 2.602 | 1.043 | .087 |
| 59**%** | 2.130 | 2.609 | 1.087 | .087 |
| 60**%** | 2.130 | 2.652 | 1.130 | .087 |
| 61**%** | 2.174 | 2.652 | 1.174 | .087 |
| 62**%** | 2.174 | 2.696 | 1.217 | .087 |
| 63**%** | 2.174 | 2.696 | 1.233 | .087 |
| 64**%** | 2.217 | 2.783 | 1.261 | .087 |
| 65**%** | 2.217 | 2.826 | 1.261 | .087 |
| 66**%** | 2.261 | 2.870 | 1.304 | .087 |
| 67**%** | 2.304 | 2.870 | 1.304 | .130 |
| 68**%** | 2.304 | 2.870 | 1.391 | .130 |
| 69**%** | 2.348 | 2.913 | 1.435 | .130 |
| 70**%** | 2.348 | 2.913 | 1.478 | .130 |
| 71**%** | 2.391 | 2.957 | 1.478 | .130 |
| 72**%** | 2.391 | 3.000 | 1.478 | .130 |
| 73**%** | 2.391 | 3.000 | 1.565 | .130 |
| 74**%** | 2.391 | 3.043 | 1.609 | .174 |
| 75**%** | 2.435 | 3.043 | 1.674 | .174 |
| 76**%** | 2.435 | 3.087 | 1.696 | .174 |
| 77**%** | 2.478 | 3.087 | 1.739 | .174 |
| 78**%** | 2.478 | 3.087 | 1.739 | .174 |
| 79**%** | 2.522 | 3.130 | 1.754 | .174 |
| 80**%** | 2.565 | 3.130 | 1.798 | .174 |
| 81**%** | 2.652 | 3.130 | 1.826 | .174 |
| 82**%** | 2.739 | 3.174 | 1.870 | .217 |
| 83**%** | 2.783 | 3.174 | 1.913 | .217 |
| 84**%** | 2.783 | 3.217 | 1.913 | .217 |
| 85**%** | 2.826 | 3.217 | 2.000 | .217 |
| 86**%** | 2.826 | 3.261 | 2.000 | .217 |
| 87**%** | 2.870 | 3.304 | 2.043 | .261 |
| 88**%** | 2.870 | 3.304 | 2.087 | .261 |
| 89**%** | 2.913 | 3.348 | 2.217 | .304 |
| 90**%** | 2.913 | 3.391 | 2.261 | .304 |
| 91**%** | 2.957 | 3.435 | 2.304 | .348 |
| 92**%** | 2.957 | 3.478 | 2.363 | .348 |
| 93**%** | 3.000 | 3.478 | 2.435 | .391 |
| 94**%** | 3.043 | 3.522 | 2.519 | .391 |
| 95**%** | 3.087 | 3.565 | 2.624 | .478 |
| 96**%** | 3.217 | 3.609 | 2.696 | .478 |
| 97**%** | 3.217 | 3.652 | 2.826 | .609 |
| 98**%** | 3.304 | 3.739 | 2.870 | .696 |
| 99**%** | 3.478 | 3.783 | 3.304 | .783 |
| 100**%** | 3.565 | 4.000 | 3.435 | 1.304 |
