# Supplementary material for: A proposed severity classification of borderline symptoms using the borderline symptom list (BSL-23)
Source: Borderline Personal Disord Emot Dysregul. 2020 Jun 1;7:11. doi: 10.1186/s40479-020-00126-6 (PMC7262769; doi:10.1186/s40479-020-00126-6)
Supplement: Supplementary file 2 — Additional file 2: Table S2. Youden’s Index and Coordinates of the ROC curve for BPD_VAL vs HC. [file 40479_2020_126_MOESM2_ESM.docx]

Supplementary table 2: Youden’s Index and Coordinates of the ROC curve for BPD_VAL vs HC.

| **Sensitivity, Specificity & Youden's Index** | | | | |
| --- | --- | --- | --- | --- |
|  | Positive if Greater Than or Equal To | Sensitivity | Specificity | Youden |
| 1 | ,0217 | 1,000 | ,331 | ,331 |
| 2 | ,0493 | 1,000 | ,534 | ,534 |
| 3 | ,0570 | 1,000 | ,537 | ,537 |
| 4 | ,0728 | 1,000 | ,539 | ,539 |
| 5 | ,1087 | 1,000 | ,660 | ,660 |
| 6 | ,1522 | ,997 | ,739 | ,736 |
| 7 | ,1957 | ,997 | ,817 | ,814 |
| 8 | ,2391 | ,994 | ,865 | ,859 |
| 9 | ,2826 | ,994 | ,888 | ,881 |
| 10 | ,3261 | ,994 | ,902 | ,895 |
| 11 | ,3696 | ,994 | ,930 | ,923 |
| 12 | ,4130 | ,987 | ,944 | ,931 |
| 13 | ,4541 | ,987 | ,949 | ,937 |
| 14 | ,4759 | ,984 | ,949 | ,934 |
| 15 | ,5000 | ,984 | ,961 | ,945 |
| 16 | ,5435 | ,978 | ,963 | ,941 |
| 17 | ,5870 | ,978 | ,966 | ,944 |
| 18 | ,6213 | ,972 | ,975 | ,946 |
| 19 | ,6430 | ,972 | ,978 | ,949 |
| 20 | ,6739 | ,965 | ,978 | ,943 |
| 21 | ,7174 | ,950 | ,980 | ,930 |
| 22 | ,7609 | ,946 | ,989 | ,935 |
| 23 | ,7902 | ,946 | ,992 | ,938 |
| 24 | ,8120 | ,943 | ,992 | ,935 |
| 25 | ,8478 | ,937 | ,992 | ,928 |
| 26 | ,8913 | ,931 | ,992 | ,922 |
| 27 | ,9348 | ,915 | ,992 | ,906 |
| 28 | ,9783 | ,915 | ,994 | ,909 |
| 29 | 1,0059 | ,909 | ,997 | ,906 |
| 30 | 1,0276 | ,905 | ,997 | ,903 |
| 31 | 1,0652 | ,902 | ,997 | ,899 |
| 32 | 1,1087 | ,899 | ,997 | ,896 |
| 33 | 1,1522 | ,893 | ,997 | ,890 |
| 34 | 1,1957 | ,880 | ,997 | ,877 |
| 35 | 1,2391 | ,877 | ,997 | ,874 |
| 36 | 1,2826 | ,861 | ,997 | ,858 |
| 37 | 1,3261 | ,855 | 1,000 | ,855 |
| 38 | 1,3567 | ,852 | 1,000 | ,852 |
| 39 | 1,3785 | ,849 | 1,000 | ,849 |
| 40 | 1,4130 | ,842 | 1,000 | ,842 |
| 41 | 1,4565 | ,833 | 1,000 | ,833 |
| 42 | 1,5000 | ,826 | 1,000 | ,826 |
| 43 | 1,5435 | ,814 | 1,000 | ,814 |
| 44 | 1,5870 | ,798 | 1,000 | ,798 |
| 45 | 1,6304 | ,789 | 1,000 | ,789 |
| 46 | 1,6739 | ,760 | 1,000 | ,760 |
| 47 | 1,6985 | ,751 | 1,000 | ,751 |
| 48 | 1,7202 | ,748 | 1,000 | ,748 |
| 49 | 1,7400 | ,738 | 1,000 | ,738 |
| 50 | 1,7476 | ,735 | 1,000 | ,735 |
| 51 | 1,7685 | ,732 | 1,000 | ,732 |
| 52 | 1,7902 | ,722 | 1,000 | ,722 |
| 53 | 1,8096 | ,719 | 1,000 | ,719 |
| 54 | 1,8237 | ,716 | 1,000 | ,716 |
| 55 | 1,8478 | ,713 | 1,000 | ,713 |
| 56 | 1,8913 | ,700 | 1,000 | ,700 |
| 57 | 1,9139 | ,688 | 1,000 | ,688 |
| 58 | 1,9357 | ,685 | 1,000 | ,685 |
| 59 | 1,9783 | ,672 | 1,000 | ,672 |
| 60 | 2,0217 | ,662 | 1,000 | ,662 |
| 61 | 2,0652 | ,650 | 1,000 | ,650 |
| 62 | 2,1087 | ,631 | 1,000 | ,631 |
| 63 | 2,1522 | ,606 | 1,000 | ,606 |
| 64 | 2,1815 | ,596 | 1,000 | ,596 |
| 65 | 2,2033 | ,593 | 1,000 | ,593 |
| 66 | 2,2391 | ,571 | 1,000 | ,571 |
| 67 | 2,2826 | ,555 | 1,000 | ,555 |
| 68 | 2,3261 | ,543 | 1,000 | ,543 |
| 69 | 2,3687 | ,530 | 1,000 | ,530 |
| 70 | 2,3904 | ,527 | 1,000 | ,527 |
| 71 | 2,3989 | ,502 | 1,000 | ,502 |
| 72 | 2,4091 | ,498 | 1,000 | ,498 |
| 73 | 2,4233 | ,495 | 1,000 | ,495 |
| 74 | 2,4565 | ,467 | 1,000 | ,467 |
| 75 | 2,5000 | ,451 | 1,000 | ,451 |
| 76 | 2,5435 | ,438 | 1,000 | ,438 |
| 77 | 2,5837 | ,423 | 1,000 | ,423 |
| 78 | 2,6054 | ,420 | 1,000 | ,420 |
| 79 | 2,6304 | ,401 | 1,000 | ,401 |
| 80 | 2,6739 | ,385 | 1,000 | ,385 |
| 81 | 2,7391 | ,366 | 1,000 | ,366 |
| 82 | 2,8043 | ,356 | 1,000 | ,356 |
| 83 | 2,8337 | ,347 | 1,000 | ,347 |
| 84 | 2,8554 | ,344 | 1,000 | ,344 |
| 85 | 2,8724 | ,319 | 1,000 | ,319 |
| 86 | 2,8783 | ,315 | 1,000 | ,315 |
| 87 | 2,8972 | ,312 | 1,000 | ,312 |
| 88 | 2,9348 | ,297 | 1,000 | ,297 |
| 89 | 2,9783 | ,281 | 1,000 | ,281 |
| 90 | 3,0217 | ,265 | 1,000 | ,265 |
| 91 | 3,0652 | ,249 | 1,000 | ,249 |
| 92 | 3,1087 | ,215 | 1,000 | ,215 |
| 93 | 3,1522 | ,189 | 1,000 | ,189 |
| 94 | 3,1957 | ,167 | 1,000 | ,167 |
| 95 | 3,2391 | ,148 | 1,000 | ,148 |
| 96 | 3,2826 | ,136 | 1,000 | ,136 |
| 97 | 3,3261 | ,117 | 1,000 | ,117 |
| 98 | 3,3596 | ,107 | 1,000 | ,107 |
| 99 | 3,3813 | ,104 | 1,000 | ,104 |
| 100 | 3,4130 | ,091 | 1,000 | ,091 |
| 101 | 3,4565 | ,088 | 1,000 | ,088 |
| 102 | 3,5000 | ,066 | 1,000 | ,066 |
| 103 | 3,5435 | ,054 | 1,000 | ,054 |
| 104 | 3,5870 | ,041 | 1,000 | ,041 |
| 105 | 3,6304 | ,035 | 1,000 | ,035 |
| 106 | 3,6739 | ,028 | 1,000 | ,028 |
| 107 | 3,7174 | ,025 | 1,000 | ,025 |
| 108 | 3,7467 | ,019 | 1,000 | ,019 |
| 109 | 3,7685 | ,016 | 1,000 | ,016 |
| 110 | 3,8043 | ,009 | 1,000 | ,009 |
| 111 | 3,9130 | ,006 | 1,000 | ,006 |
