# Supplementary material for: A proposed severity classification of borderline symptoms using the borderline symptom list (BSL-23)
Source: Borderline Personal Disord Emot Dysregul. 2020 Jun 1;7:11. doi: 10.1186/s40479-020-00126-6 (PMC7262769; doi:10.1186/s40479-020-00126-6)
Supplement: Supplementary file 3 — Additional file 3: Table S3. Youden’s Index and Coordinates of the ROC curve for BPD_VAL vs CC. [file 40479_2020_126_MOESM3_ESM.docx]

Supplementary table 3: Youden’s Index and Coordinates of the ROC curve for BPD_VAL vs CC.

| **Sensitivity, Specificity & Youden's Index** | | | | |
| --- | --- | --- | --- | --- |
|  | Positive if Greater Than or Equal To | Sensitivity | Specificity | Youden |
| 1 | ,0217 | 1,000 | ,011 | ,011 |
| 2 | ,0507 | 1,000 | ,023 | ,023 |
| 3 | ,0691 | 1,000 | ,028 | ,028 |
| 4 | ,0837 | 1,000 | ,034 | ,034 |
| 5 | ,1087 | 1,000 | ,063 | ,063 |
| 6 | ,1522 | ,997 | ,074 | ,071 |
| 7 | ,1957 | ,997 | ,085 | ,082 |
| 8 | ,2274 | ,994 | ,102 | ,096 |
| 9 | ,2491 | ,994 | ,108 | ,102 |
| 10 | ,2685 | ,994 | ,142 | ,136 |
| 11 | ,2902 | ,994 | ,148 | ,141 |
| 12 | ,3120 | ,994 | ,199 | ,193 |
| 13 | ,3337 | ,994 | ,205 | ,198 |
| 14 | ,3696 | ,994 | ,227 | ,221 |
| 15 | ,4130 | ,987 | ,227 | ,215 |
| 16 | ,4541 | ,987 | ,244 | ,232 |
| 17 | ,4759 | ,984 | ,250 | ,234 |
| 18 | ,5000 | ,984 | ,267 | ,251 |
| 19 | ,5435 | ,978 | ,290 | ,268 |
| 20 | ,5748 | ,978 | ,301 | ,279 |
| 21 | ,5865 | ,978 | ,307 | ,285 |
| 22 | ,5987 | ,978 | ,313 | ,290 |
| 23 | ,6163 | ,972 | ,347 | ,318 |
| 24 | ,6380 | ,972 | ,358 | ,330 |
| 25 | ,6739 | ,965 | ,381 | ,346 |
| 26 | ,6965 | ,950 | ,409 | ,359 |
| 27 | ,7041 | ,950 | ,415 | ,364 |
| 28 | ,7250 | ,950 | ,420 | ,370 |
| 29 | ,7467 | ,946 | ,449 | ,395 |
| 30 | ,7685 | ,946 | ,455 | ,401 |
| 31 | ,7902 | ,946 | ,472 | ,418 |
| 32 | ,8120 | ,943 | ,472 | ,415 |
| 33 | ,8478 | ,937 | ,500 | ,437 |
| 34 | ,8913 | ,931 | ,511 | ,442 |
| 35 | ,9167 | ,915 | ,528 | ,443 |
| 36 | ,9243 | ,915 | ,534 | ,449 |
| 37 | ,9424 | ,915 | ,545 | ,460 |
| 38 | ,9783 | ,915 | ,551 | ,466 |
| 39 | 1,0059 | ,909 | ,563 | ,471 |
| 40 | 1,0276 | ,905 | ,563 | ,468 |
| 41 | 1,0652 | ,902 | ,585 | ,487 |
| 42 | 1,1087 | ,899 | ,597 | ,496 |
| 43 | 1,1422 | ,893 | ,602 | ,495 |
| 44 | 1,1639 | ,893 | ,608 | ,501 |
| 45 | 1,1957 | ,880 | ,619 | ,499 |
| 46 | 1,2250 | ,877 | ,625 | ,502 |
| 47 | 1,2443 | ,877 | ,631 | ,508 |
| 48 | 1,2585 | ,877 | ,636 | ,513 |
| 49 | 1,2826 | ,861 | ,659 | ,520 |
| 50 | 1,3261 | ,855 | ,670 | ,525 |
| 51 | 1,3567 | ,852 | ,676 | ,528 |
| 52 | 1,3785 | ,849 | ,676 | ,525 |
| 53 | 1,4130 | ,842 | ,688 | ,530 |
| 54 | 1,4552 | ,833 | ,693 | ,526 |
| 55 | 1,4770 | ,833 | ,699 | ,532 |
| 56 | 1,5000 | ,826 | ,722 | ,548 |
| 57 | 1,5435 | ,814 | ,727 | ,541 |
| 58 | 1,5870 | ,798 | ,739 | ,537 |
| 59 | 1,6304 | ,789 | ,744 | ,533 |
| 60 | 1,6739 | ,760 | ,750 | ,510 |
| 61 | 1,6985 | ,751 | ,767 | ,518 |
| 62 | 1,7202 | ,748 | ,767 | ,515 |
| 63 | 1,7400 | ,738 | ,784 | ,522 |
| 64 | 1,7476 | ,735 | ,784 | ,519 |
| 65 | 1,7685 | ,732 | ,795 | ,527 |
| 66 | 1,7902 | ,722 | ,795 | ,518 |
| 67 | 1,8096 | ,719 | ,801 | ,520 |
| 68 | 1,8237 | ,716 | ,801 | ,517 |
| 69 | 1,8478 | ,713 | ,813 | ,525 |
| 70 | 1,8772 | ,700 | ,824 | ,524 |
| 71 | 1,8989 | ,700 | ,830 | ,530 |
| 72 | 1,9139 | ,688 | ,841 | ,529 |
| 73 | 1,9357 | ,685 | ,841 | ,525 |
| 74 | 1,9783 | ,672 | ,847 | ,519 |
| 75 | 2,0217 | ,662 | ,864 | ,526 |
| 76 | 2,0652 | ,650 | ,875 | ,525 |
| 77 | 2,1087 | ,631 | ,881 | ,512 |
| 78 | 2,1522 | ,606 | ,886 | ,492 |
| 79 | 2,1815 | ,596 | ,886 | ,483 |
| 80 | 2,2033 | ,593 | ,886 | ,479 |
| 81 | 2,2391 | ,571 | ,898 | ,469 |
| 82 | 2,2698 | ,555 | ,903 | ,459 |
| 83 | 2,2915 | ,555 | ,909 | ,464 |
| 84 | 2,3261 | ,543 | ,915 | ,457 |
| 85 | 2,3554 | ,530 | ,915 | ,445 |
| 86 | 2,3763 | ,530 | ,920 | ,450 |
| 87 | 2,3904 | ,527 | ,920 | ,447 |
| 88 | 2,3989 | ,502 | ,926 | ,428 |
| 89 | 2,4091 | ,498 | ,926 | ,425 |
| 90 | 2,4233 | ,495 | ,926 | ,421 |
| 91 | 2,4565 | ,467 | ,932 | ,399 |
| 92 | 2,4987 | ,451 | ,938 | ,389 |
| 93 | 2,5204 | ,451 | ,943 | ,394 |
| 94 | 2,5435 | ,438 | ,943 | ,382 |
| 95 | 2,5837 | ,423 | ,949 | ,372 |
| 96 | 2,6054 | ,420 | ,949 | ,368 |
| 97 | 2,6163 | ,401 | ,949 | ,349 |
| 98 | 2,6380 | ,401 | ,955 | ,355 |
| 99 | 2,6739 | ,385 | ,955 | ,339 |
| 100 | 2,7174 | ,366 | ,960 | ,326 |
| 101 | 2,7609 | ,366 | ,966 | ,332 |
| 102 | 2,8043 | ,356 | ,966 | ,322 |
| 103 | 2,8337 | ,347 | ,977 | ,324 |
| 104 | 2,8554 | ,344 | ,977 | ,321 |
| 105 | 2,8724 | ,319 | ,983 | ,302 |
| 106 | 2,8783 | ,315 | ,983 | ,298 |
| 107 | 2,8972 | ,312 | ,983 | ,295 |
| 108 | 2,9348 | ,297 | ,983 | ,279 |
| 109 | 2,9783 | ,281 | ,983 | ,264 |
| 110 | 3,0217 | ,265 | ,989 | ,254 |
| 111 | 3,0652 | ,249 | ,989 | ,238 |
| 112 | 3,1087 | ,215 | ,989 | ,203 |
| 113 | 3,1522 | ,189 | ,989 | ,178 |
| 114 | 3,1957 | ,167 | ,989 | ,156 |
| 115 | 3,2391 | ,148 | ,989 | ,137 |
| 116 | 3,2826 | ,136 | ,989 | ,124 |
| 117 | 3,3261 | ,117 | ,994 | ,111 |
| 118 | 3,3596 | ,107 | ,994 | ,102 |
| 119 | 3,3813 | ,104 | ,994 | ,098 |
| 120 | 3,4130 | ,091 | ,994 | ,086 |
| 121 | 3,4565 | ,088 | 1,000 | ,088 |
| 122 | 3,5000 | ,066 | 1,000 | ,066 |
| 123 | 3,5435 | ,054 | 1,000 | ,054 |
| 124 | 3,5870 | ,041 | 1,000 | ,041 |
| 125 | 3,6304 | ,035 | 1,000 | ,035 |
| 126 | 3,6739 | ,028 | 1,000 | ,028 |
| 127 | 3,7174 | ,025 | 1,000 | ,025 |
| 128 | 3,7467 | ,019 | 1,000 | ,019 |
| 129 | 3,7685 | ,016 | 1,000 | ,016 |
| 130 | 3,8043 | ,009 | 1,000 | ,009 |
| 131 | 3,9130 | ,006 | 1,000 | ,006 |
